# Design and Validation of an Augmented Reality System for Laparoscopic Surgery in a Real Environment

**DOI:** 10.1155/2013/758491

**Published:** 2013-10-23

**Authors:** F. López-Mir, V. Naranjo, J. J. Fuertes, M. Alcañiz, J. Bueno, E. Pareja

**Affiliations:** ^1^Instituto Interuniversitario de Investigación en Bioingeniería y Tecnología Orientada al Ser Humano (I3BH), Universitat Politècnica de València, I3BH/LabHuman, Camino de Vera s/n, 46022 Valencia, Spain; ^2^CIBER, Fisiopatología de Obesidad y Nutrición (CB06/03), Instituto de Salud Carlos III, 28029 Madrid, Spain; ^3^Unidad de Cirugía Hepatobiliopancreática y Trasplante Hepático, Hospital Universitario y Politécnico La Fe, 46026 Valencia, Spain

## Abstract

*Purpose*. This work presents the protocol carried out in the development and validation of an augmented reality system which was installed in an operating theatre to help surgeons with trocar placement during laparoscopic surgery. The purpose of this validation is to demonstrate the improvements that this system can provide to the field of medicine, particularly surgery. *Method*. Two experiments that were noninvasive for both the patient and the surgeon were designed. In one of these experiments the augmented reality system was used, the other one was the control experiment, and the system was not used. The type of operation selected for all cases was a cholecystectomy due to the low degree of complexity and complications before, during, and after the surgery. The technique used in the placement of trocars was the French technique, but the results can be extrapolated to any other technique and operation. *Results and Conclusion*. Four clinicians and ninety-six measurements obtained of twenty-four patients (randomly assigned in each experiment) were involved in these experiments. The final results show an improvement in accuracy and variability of 33% and 63%, respectively, in comparison to traditional methods, demonstrating that the use of an augmented reality system offers advantages for trocar placement in laparoscopic surgery.

## 1. Introduction

Laparoscopic surgery has proven to be an alternative to traditional open surgery since smaller incisions are made in the abdomen of the patient [1]. The laparoscopic camera and the different endoscopic instruments are introduced through trocars, the hollow cylindrical instruments that are placed into these incisions. Thanks to these smaller incisions, this surgery offers many advantages to the patient, such as less chance of infection, less subsequent operations to remake the abdominal muscle, and so forth. Consequently, the recovery time for the patient is faster both physically and psychologically, which means a lower postoperative cost for the hospital [2].

The main drawbacks of laparoscopic surgery in contrast to open surgery are the lack of direct vision, the need for hand-eye coordination, and the lack of tactile feedback to the surgeon. Another problem is related to the trocar placement since it may result in more invasive surgery. Currently, the incisions are made by palpation based on the experience and skill of the surgeon. The improper placement of trocars in an operation, such as in the lymph node dissection in the hepatoduodenal ligament, can complicate the operation. In these cases, a relocation of the trocar might be necessary (and more incisions than strictly necessary will be made) thereby limiting the advantages of laparoscopic surgery mentioned above [3].

Augmented reality (AR) is a 3D computer vision technique that is characterized by the real-time fusion of virtual elements on a real space [4]. Currently, augmented reality offers enormous potential in many fields such as education, simulation, architecture, advertising, navigation devices, medicine, and rehabilitation [4]. Surgery is the branch of medicine where augmented reality has more potential for application because it can provide surgeons with preoperative information (magnetic resonance imaging or MRI, radiography, 3D reconstructions, etc.) in the same place and at the same time that they are operating. Thus, some of the drawbacks previously cited are alleviated [5, 6].

In [7], a taxonomy of augmented reality systems in image-guided surgery is proposed. The work compares and analyzes several systems which use augmented reality technology in surgery applications. The analysis is based on the type of input data, the visualization format, and the way in which data is displayed in the operating theatre. The objectives of this comparison are to establish the syntax for defining a system of these characteristics and to show the principal components of an AR system for image-guided applications. In our case, following the analysis suggested in the work [7], three components have been chosen.

- Specific data of the patient: our system uses MRI of the patient and generates a 3D model of internal structures.
- A visualization format based on color coding for anatomical structures: transparency has been used in the models to give more realistic depth in the virtual model.
- A full HD monitor for displaying data.

**Figure 1:**
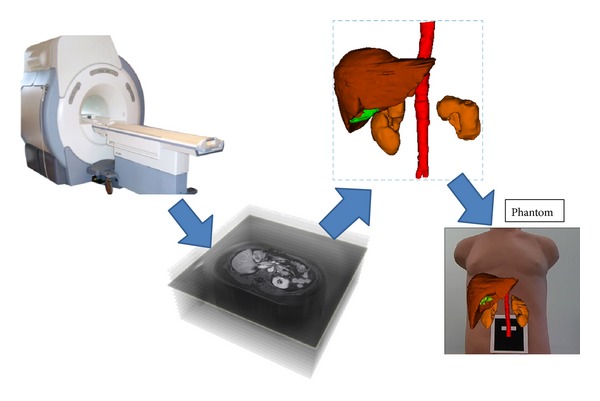
Magnetic resonance machine. Magnetic resonance images and 3D model of abdominal organs. Registration and fusion with real-time video in a phantom.

The main limitation of AR systems is the registration technique employed. In our case, the registration and fusion are done between a 3D volume from the segmented magnetic resonance images of the patient and the real-time image that is recorded by a webcam placed over the patient in the operating theater, specifically above of the patient's abdomen (Figure 1).

Some authors have developed techniques to improve and automate preoperative placement of trocars. Based on 3D information extracted from computed tomography (CT) images or MRI, the surgeons must remember this information once they are in the operating theatre. In [8], an optimal access system with virtual endoscopic views is proposed, making the simulation with a phantom. In [9, 10], the problem is addressed in image-guided surgery, and trocar placement is optimized from a robotic point of view. The validation is performed on animals. In [11], the system requires the use of fiducials that have to be in the same position as when the CT was acquired. In addition, the position and orientation of the patient have to be the same in the operating theater.

Other authors deal with the problem in the operating theater similarly to the method presented in this work, but they are focused on the navigation during the intervention using the image that the endoscopic camera provides. In [12], a registration with fiducials is carried out to monitor the camera. These fiducials must be placed over the patient in the same positions in the operating theater and when the CT is acquired. In [13], 3D information is merged into the laparoscopic video. In [14], the validation was done in animals, and the registration and fusion processes were done manually thanks to the surgeon's anatomical knowledge. In [15], a head-mounted display is used, and the validation was carried out in a commercial phantom.

The experiments carried out in this work were performed in laparoscopic cholecystectomies. This type of intervention is a common solution for diseases such as symptomatic gallstones [16]. It is a common operation with low probability of pre- and postoperative complications. The placement of trocars is usually performed using either French or American techniques [17]. The choice of one technique or another does not determine the outcome of the experiments, which can be extrapolated from each other. For our experiments, the surgeons chose the French technique because they are accustomed to using it. Both techniques are based on placing four abdominal trocars. Three of them are placed in the same positions in both cases; the fourth trocar differs in the American and the French technique from the area below the sternum in the American and in the opposite side of the liver in the French. In both cases, the surgeon draws four marks with a biocompatible pen taking into account external anatomical references. These marks serve as the initial references of where to make the final incisions. The first trocar (which is called the Veress needle or Hasson cannula and is different from others) is always located at the same position that has been marked with the pen (one centimeter above the navel after making an incision of about 10 mm with the scalpel). By means of this trocar, the pneumoperitoneum technique is performed, and the abdominal cavity is deformed [18]. Subsequently, the endoscopic camera is inserted through this same trocar to visualize the abdominal cavity (keeping the insufflator hose connected to maintain pneumoperitoneum throughout the entire surgery). The other three incisions are made with the internal vision of the camera and palpation, correcting the position of the marks made with the pen. For two of the incisions, the primary surgeon inserts the surgical instruments (scalpel, forceps, scissors, etc.), and the other incision is used by the secondary surgeon according to the principal surgeon instructions.

The goodness of our system using augmented reality in the operating theatre was determined by measuring the precision offered by the system compared to not using it. Four distances relating to the four incisions made in the patient (||*d*_*i*_||, *i* = {1, 2, 3, 4})  were obtained. In this work, the position of the incision (*P*_i_* = 〈*x*_i_*, *y*_i_*, *z*_i_*〉)  is equal to the position of the pen mark (*P*_*i*_ = 〈*x*_*i*_, *y*_*i*_, *z*_*i*_〉) plus an offset (*d*_*i*_ = 〈*d*_*xi*_, *d*_*yi*_, *d*_*zi*_〉) (1). The measured distance $||d_{i}||=\sqrt{d_{xi}^{2}+d_{yi}^{2}{+d}_{zi}^{2}}$, *i* = {1, 2, 3, 4} is due to different factor such as displacements and deformities of the pneumoperitoneum technique (*d*_neum_), the distinctive features of the internal anatomy of the patient (*d*_patient_) that the surgeon does not notice at the time of making the marks with the pen, and the experience and skill of the surgeon (*d*_surgeon_):

(1) $$\begin{array}{c} P_{i}^{*}=P_{i}+d_{i}, \end{array}$$

(2) $$\begin{array}{c} d_{i}=d_{\text{neum}}+d_{\text{patient}\;}+d_{\text{surgeon}}+d_{\text{other}\;}. \end{array}$$

The distance *d*_other_ is related to any error not taking into account by the other variables, for example, operating theatre characteristics (light, position of the patient in the stretcher). When an augmented reality system is used, *d*_*i*_ may be decomposed into the elements of (3) (similar to (2), but decomposing *d*_other  _ into three new corrections). The augmented reality system introduces an error due to the applied registration method (*d*_ra_) which is related to the precision offered by the AR system (Section 2.2.1). There is also another error related to the accuracy in the segmentation procedure (*d*_segment_) of the 3D model which will be projected onto the patient. In our experiments, this segmentation was previously done by an expert and then reviewed by a second expert to check it. This assumption leads us to conclude that the segmentation error can be considered zero or equal to the pixel resolution. The distance *d*_other_′ is similar to *d*_other_ (and other errors as the manual alignment of the marker are also included in this distance). In any case, the hypothesis of this work is that the errors introduced by the augmented reality system will be compensated by the global improvement in *d*_*i*_:

(3) $$\begin{array}{c} d_{i}=d_{\text{neum}}+d_{\text{patient}\;}+d_{\text{surgeon}}+d_{\text{ra}}+d_{\text{segment}}+d_{\text{other}}^{\prime }. \end{array}$$

The purpose of this work is to measure the ||*d*_*i*_|| distance for the four trocars which *P*_i_* (incisions where the trocar is inserted) and *P*_*i*_ (marks made with the pen) are known. These distances are measured in some patients when the system is used and on other patients when the system is not used. The goal is to verify if the use of augmented reality system minimizes ||*d*_*i*_||.

Several authors have attempted to measure the error caused by an augmented reality system in an operating theater. Most validate it on phantoms and in the maxillofacial and neurosurgery fields. The high resolution of these images and the rigidity of these structures indicate that this error can be explained mainly by (but not limited to) a registration error associated with augmented reality algorithms [19–26]. This error is measured qualitatively [22] or quantitatively as being on the order of several millimeters. Other authors validate their algorithms using abdominal operations. In [27], an AR system is applied in a liver phantom limiting the measured error. The AR system presented in [11] is validated for liver surgery on pigs where registration with 4 fiducials is used to measure its accuracy.

The rest of this paper is organized as follows.  Section 2 is divided into two parts, the first part explains the AR system, and the second part describes the protocol of the experiments that were carried out.  Section 3 presents the results, and Section 4 presents conclusions and discussions. The primary contribution of this paper is the design, performance, and validation of an augmented reality system, the ergonomic study of the visualization devices and the protocol definition for its validation on real patients in an operating theatre.

## 2. Methodology

### 2.1. Augmented Reality System

#### 2.1.1. Virtual 3D Model

**Figure 2:**
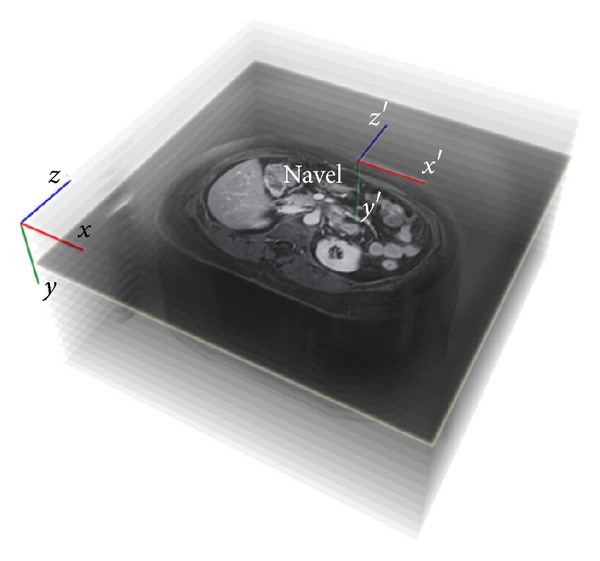
Change in the coordinate system.

When MR images are acquired, the patient must lie on a stretcher with his/her back straight and centered on both sides to calculate the position and the orientation relative to an initial coordinate system. A virtual model of the patient's organs is extracted from these images using techniques of digital image processing, especially our own image segmentation algorithms [28] and others developed in [29]. With this model, the clinician selects the patient's navel in the MRI images to establish the origin of 3D space at that point in order to perform the registration with the real-time image (4). The new coordinate system is

(4) $$\begin{array}{c} x^{\prime }=\alpha +x,\;\;y^{\prime }=\beta +y,\;\;z^{\prime }=\gamma +z, \end{array}$$

where *α*, *β*, and *γ* are the coordinates of the center of the patient's navel with respect to the initial coordinate system (*x*, *y*, *z*), (Figure 2).

#### 2.1.2. Camera Calibration and Real-Time Image

**Figure 3:**
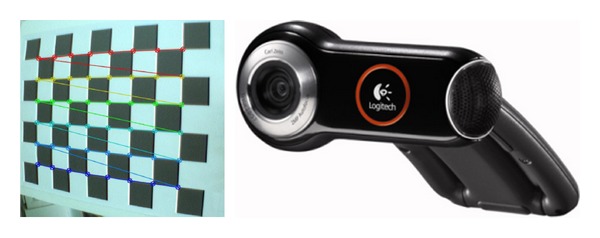
Checkerboard that is used to calibrate the camera. A Logitech QuickCam Pro 9000 webcam.

The real-time images are recorded with a camera that shows the area of interest throughout the entire surgery. Initially, the intrinsic parameters of the camera are obtained to calibrate it. To do this, it is necessary to have different captures of planar checkerboard patterns (see Figure 3), which should be different for each calibration image. Zhang's method is used for the calibration step, taking the correspondence between 2D image points and 3D scene points over a number of images [30].

The 3 × 3 intrinsic matrix *K* and the vector *γ* of the camera with the distortion parameters have the following form:

(5) $$\begin{array}{c} K=\left[\begin{array}{ccc} f & s & u\\ 0 & af & v\\ 0 & 0 & 1 \end{array}\right],\;\;\gamma =\left[\begin{array}{c} \alpha_{1}\\ \begin{array}{c} \alpha_{2}\\ \begin{array}{c} \beta_{1}\\ \beta_{2} \end{array} \end{array} \end{array}\right], \end{array}$$

where *f* is the focal length, (*u*, *v*) is the optical center of the camera, *a* is the aspect ratio, *s* is the camera skew between the *x*- and *y*-axes, *α*_1_ and *α*_2_ are the radial distortion parameters, and *β*_1_ and *β*_2_ are the tangential distortion parameters. The values of these parameters for camera calibration were (all in mm)

(6) K=[518.550349.72701.33∗517.48279.897001],γ=[−0.3232760.112309−0.000341309−0.00175445].

**Figure 4:**
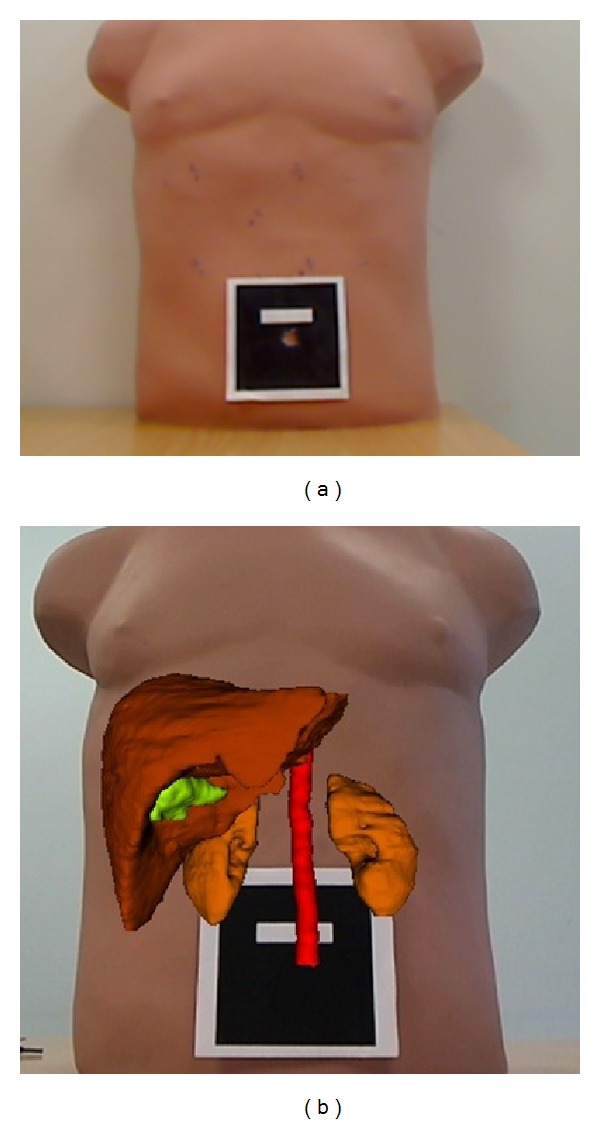
(a) Hexadecimal binary mark and (b) fusion of virtual model in a phantom.

Then, a hexadecimal mark is placed on the navel and centered and oriented as shown in Figure 4. It is advisable to keep the camera parallel to the patient's trunk in order to improve the accuracy of the system, but it is not mandatory (as explained in Section 2.1.3) because the system takes into account the inclination between the patient and the camera position. The next steps are the hexadecimal mark detection and the registration and fusion of the real image with the virtual model of the patient.

#### 2.1.3. Registration, Fusion, and Hexadecimal Mark Detection

A binary hexadecimal code marker of 8.45 × 8.45 centimeters is used in this step. First, the RGB captured image is converted to a binary image, and the edge of the marker is detected thanks to an adaptive threshold algorithm based on the technique of Pintaric [31]. Basically, “*this technique evaluates the mean pixel luminance over a thresholding region of interest, which is defined as a bounding rectangle around the marker axis-aligned corner vertices in the screen-space.*”

Afterwards, the relative marker position and orientation with respect to the camera (view point) can also be estimated from a planar structure when the internal parameters are known, in order to apply them to the virtual model. First, a 3D/2D homography matrix must be calculated to later obtain the projective matrix, as detailed in [32].

A 3D/2D correspondence (*m*, *M*) includes a 3D point *M* and a 2D pixel point *m*, which are represented as (*X*, *Y*, *Z*, 1) and (*x*,*y*,1)^*T*^, respectively. (*m*, *M*) is related by the 3 × 3 projective matrix *P*_*i*_ as [33] shows:

(7) $$\begin{array}{c} m=\lambda_{i}P_{i}M,\;P_{i}=K\left[R_{i}\;|\;t_{i}\right], \end{array}$$

where *R*_*i*_ is a 3 × 3 rotation matrix, *t*_*i*_ is the translation vector of the camera, and *λ*_*i*_ is the homogeneous scale factor that is dependent on *P*_*i*_ *M*. Specifically, considering the *z* = 0 plane, the expression of the homography that maps a point onto this plane, and its corresponding 2D point *m* under the perspective can be recovered by writing

(8) [xy1]=m=λiPiM=λiK(R1R2R3t)[XY01]=λiK(R1R2t)[XY1],

where *R*_1_, *R*_2_, and *R*_3_ are the columns of the matrix *R*. Thus, (*m*, *M*) is related by a 3 × 3 matrix *H*_*w*_^*i*^ that is called homography matrix:

(9) $$\begin{array}{c} \left[\begin{array}{c} x\\ y\\ 1 \end{array}\right]=\lambda_{i}H_{w}^{i}\left[\begin{array}{c} X\\ Y\\ 1 \end{array}\right],\;H_{w}^{i}=K\left(R_{1}R_{2}t_{i}\right). \end{array}$$

Conversely, once *H*_*w*_^*i*^  and *K* are known, the patient's pose can be recovered from (7) and (9), because *R* is a unit orthogonal matrix, as is explained in [34] (“the last column *R*_3_ is given by the cross-product *R*_1_ × *R*_2_”):

(10) $$\begin{array}{c} {K^{-1}H}_{w}^{i}=\left(R_{1}R_{2}t_{i}\right),\;\;P_{i}=K\left(R_{1}R_{2}R_{3}t_{i}\right). \end{array}$$

Generally, the patient's pose can be refined by nonlinear minimization, since the anterior processes are sensitive to noise, and, therefore, a lack of precision and the “jitter” phenomenon are produced.

In this case, the sum of the reprojection errors is minimized, which is the squared distance between the projection of the 3D points and their measured 2D coordinates. We can therefore write that

(11) $$\begin{array}{c} \left[R_{i}\;|\;t_{i}\right]=\underset{\left[R_{i}{\;|\;t}_{i}\right]}{\text{argmin}}\sum_{j}||{\text{PM}}_{j}-m_{j}||. \end{array}$$

This equation will be solved using the Levenberg-Marquardt (LM) algorithm proposed by [35], providing a solution for the problem “Nonlinear Least Squares Minimization.”

In this way, the 3D virtual model and the patient's image can be registered and fused. Just then, it is important for the patient to maintain his/her position to avoid possible registration errors.

### 2.2. Experimentation

#### 2.2.1. Error Introduced by the AR System

Before the system was validated by the surgeon in the hospital, to test how the AR module works and to determine its accuracy (*d*_ra_), the following experiment was performed. Initially, 512 × 512 CT images with a spacing-resolution of 0.488 × 0.488 × 0.625 mm per pixel were extracted from a jar by means of a GE LightSpeed VCT-5124069 machine. The model used was a 500 mL DURAN GLS 80 jar with a diameter of 101 mm. The 3D virtual model was obtained by applying a region growing algorithm taking the pixels between thresholds 150 and 2200 Hounsfield Units (HU).

**Figure 5:**
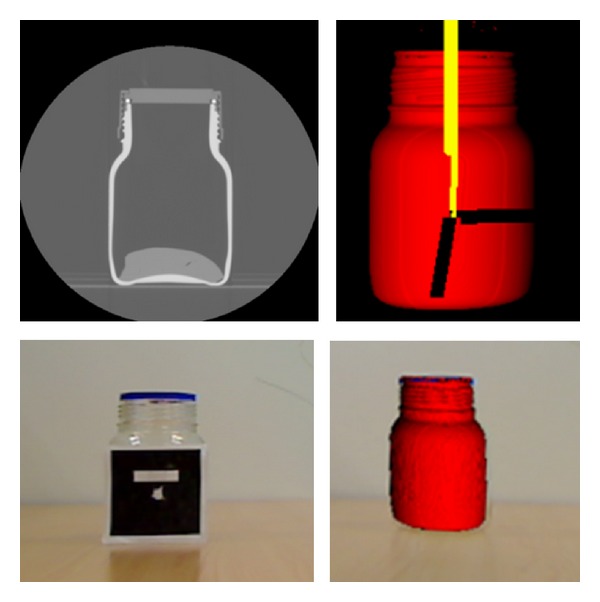
CT Jar images, 3D Jar model, marker, and registration and fusion of 3D virtual jar and real jar.

The camera was placed at a 90° degree angle relative to the real jar. Then, the middle point of the jar was selected in the CT images as the new origin, and the marker was centered on the jar. The registration and fusion were performed at that moment, taking an image of the real jar and the virtual jar to validate the system's accuracy. A full graphic example of the experiment is shown in Figure 5.

Different positions of the camera and measures were taken. Finally, it was proved that if the camera was placed at a 90° degree angle relative to the real jar, the system was introduced an error of 3 pixels (the minima of all cases). The real width of the jar and the image width provided by the camera were known, so a direct correspondence was made, and a measurement of *d*_ra_ = 2.91 mm, was obtained [26]. As mentioned in the introduction, the augmented reality systems introduce an error in the virtual pose calculation with respect to the real space. This error is irrelevant in most of the domains where an augmented reality system is used; however, it is of great importance in medicine. The main causes of this error (but not the only ones) are related to the following: the camera (the internal configuration and different lighting conditions that produce different behaviors), the accuracy of registration algorithms, and the accuracy of segmentation methods. Since it is difficult to give the results for each of these errors separately, this measure is usually given as a whole, and in our case it is defined as *d*_ra_.

#### 2.2.2. Real Patient Experiments

We carried out two experiments out on real patients. All the documents required for the adoption of the experiments were presented to the research ethics committee of the hospital. This documentation included the following.

- A certificate of commitment related to the ethical principles of clinical trials: it includes the fundamental human rights and ethical principles related to biomedical research on humans of the Helsinki and Tokyo declaration.
- A certificate of commitment from the researchers that take part in these experiments: in this certificate, the researchers agree to follow the rules and the protocol approved by the research ethics committee of the hospital.
- Informed consent, which is delivered to the patient: this document explains the purpose of study, the procedure, confidentiality, the cost, and the right to leave the study at any time without their final treatment being affected.
- A manual of the developed research: this document specifies the sample selection, the protocol used for the randomization of the sample into the two experiments, the protocol of the whole experiment, and the collection and analysis of the data.
- A validation and data collection protocol: this document has the templates and protocols necessary for the data collection of these studies.
- A request to the hospital committee for approval of the protocol: this document summarizes all the information explained above and is mandatory in order to apply for approval of the experiments.

Initially, the experiments were to be performed through a segmentation of gadolinium contrast MR images. The use of this agent improves the image contrast and facilitates the segmentation of different organs to extract the patient's 3D model. Even though it is safe, there is always the possibility of small allergic reactions in the patient. For this reason and since this contrast agent is not commonly used for this type of pathology, the committee rejected its use in the MRI acquisition. This change caused more difficulties in the segmentation procedure of abdominal organs, but it did not affect the results or conclusions of the experiments. After making this change, the clinical research committee approved the study, and the experiments were carried out.

In the first experiment, the augmented reality system is not used. The selected sample consists of 12 patients chosen randomly (eight women and four men). The following protocol was used.

**Figure 6:**
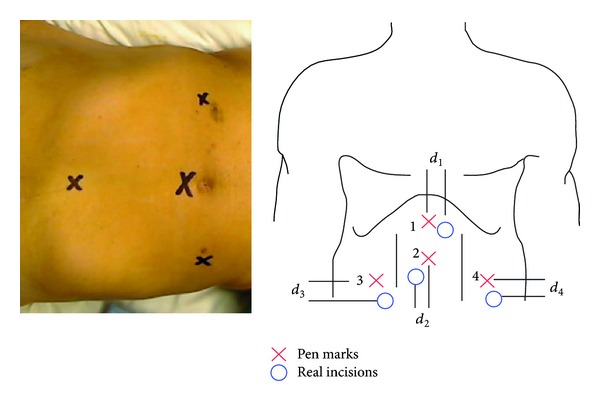
Marks in a simulation patient and marks made with the biocompatible pen (red) and real incisions (blue).

- Before the operation (the first time the surgeon visits the patient), the informed consent approved by the research ethics committee of the hospital and common information related to the MRI exam are given to the patient.
- The day of the surgery, the patient goes to the presurgery room and then passes to the operating theater.
- The surgeon performs the usual protocol until the operation ends. This protocol can be summarized as follows.
  1. First, with a biocompatible pen, the surgeon marks the points where he/she will make the four incisions through which trocars will be inserted (Figure 6, left).
  2. Second, the surgeon performs the four incisions based on his/her skill, experience, and traditional palpation techniques as explained in Section 1 (Figure 6, right).
  3. When the four trocars are placed, the surgeon begins the operation according to the specific protocol for this type of surgery.
  4. Once the gallbladder has been extracted and the four incisions are sutured, the surgeon measures the four values or distances *d*_*i*_ (Figure 6, right). These four distances measure the difference between the initial pen marks and the real incisions or, in other words, the correction that has to be made for the technique of pneumoperitoneum, the anatomical differences of patients, and the skill of the surgeon.
  5. Finally, the four incisions are bandaged.
- The surgery ends, and the patient leaves the operating theatre and goes to the postoperation room where he/she wakes up and continues with the recovery protocol.

**Figure 7:**
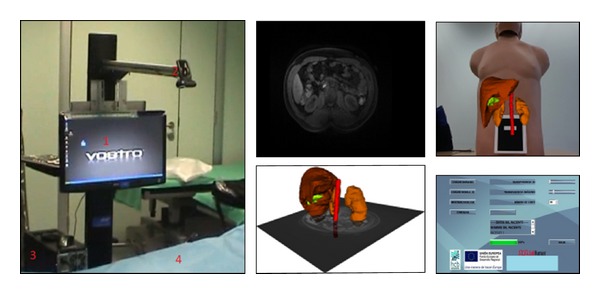
Hardware device and software system.

In the second experiment, the augmented reality system was used. The system hardware, as shown in Figure 7 (left), is composed of a display device and a camera. The goal of the camera is to capture the image in real-time in order to register and merge this sequence with the 3D virtual model of the patient. The display device is responsible for showing the fusion of the video and the virtual object. In this experiment, different display devices were evaluated.  Table 1 summarizes the advantages and the disadvantages that different displays offer; this information was obtained by a usability study taking in account operating theatre restrictions.

We chose a 23 inch full HD monitor as the display device based on the criteria of minimal interaction with the patient, minimal discomfort to the surgeon, and low cost. A dual core i3 computer with graphics card “Nvidia GT 240” was used. The screen and the camera were mounted on a stand as shown in Figure 7 (left). The stretcher with the patient was positioned between the stand and the surgeon. The actual image of the abdomen of the patient was captured by the camera which was positioned perpendicularly to the patient as shown in Figure 7.

The software (Figure 7, right) loads preoperative imaging and 3D models. A mark is placed in the navel for registration and fusion, and the final result is shown in one window of the software [26].

The sample selected for this experiment also consisted of 12 patients chosen randomly (seven men and five women). The protocol used was similar to the one used in Experiment  1.

**Figure 8:**
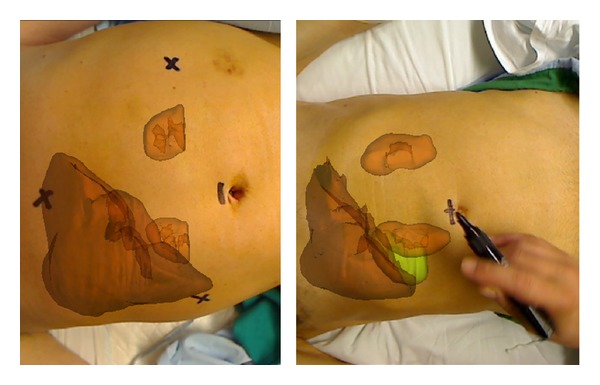
Performing the marks in two different simulation patients.

**Figure 9:**
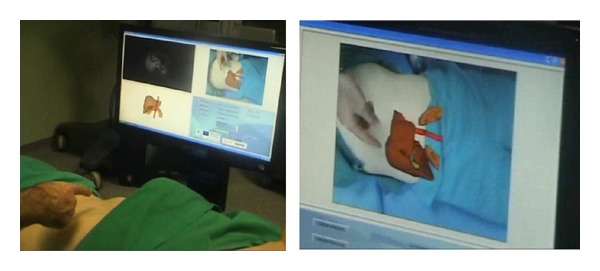
Surgeon view and image fusion in a real environment with a simulation patient.

- Before the operation, the same informed consent as in the first experiment is given to the patient. Then, the MRI is acquired.
- Thanks to different segmentation algorithms, a 3D model of the patient's organs is obtained with the MR images. Specifically, in all cases, the liver and kidneys were segmented; in some cases the gallbladder and aorta were extracted (for surgeon requirements). The tool to perform the segmentation was made ad hoc [28, 29].
- On the day of the surgery, all the steps were similar to the first experiment, with only one difference: when the surgeon marks with the pen (Figure 8), he/she used the AR system that registers and merges the 3D model with the real-time image (Figure 9). The result of this process is shown on the screen that is directly in front of the surgeon.
- Once the 4 marks are drawn, the system is removed, and the surgeon continues the usual protocol until the surgery ends.
- Finally, the same four values or distances (*d*_*i*_) as in the first experiment are measured, and the patient goes to the postoperation room to wake up and continue the recovery protocol.

## 3. Results

Ninety-six distances/measures were obtained (four per patient) half of them using the system and the other half without it. The protocol described in both experiments has been followed without major problems. The usual procedure for cholecystectomy surgery was only to be modified when the four distances were measured, after the operation had been completed and before the incisions were bandaged. If any unexpected complication appeared, these distances would not be measured in the patient. During the twenty-four surgeries, no complications occurred, so the measures were taken in all cases.  Table 2 shows the mean and standard deviation of the four distances measured in Experiment  1 on twelve patients. In this case, the traditional procedures (palpation and the skill of the surgeon) were used in the placement of trocars.

Table 3 shows the mean and standard deviation of the twelve cases in Experiment  2, that is, when the augmented reality system was used (a new procedure was added to the traditional protocol).

The Mann-Whitney *U* test was used to validate the null hypothesis of equal medians at the default 5% significance level relating to the distance measures of both experiments. A *P* value higher than 0.05 indicates that there is a nonsignificance difference, and therefore, the measures can be added. In Experiment  1, the *P* values between the four distances were *P*_*d*1–*d*3_ = 0.56, *P*_*d*1–*d*4_ = 0.93, *P*_*d*3-*d*4_ = 0.5, *P*_*d*1-*d*2_ = 0.003, *P*_*d*3-*d*2_ = 0.048, and *P*_*d*4–*d*2_ = 0.03. If these values are analyzed the distance *d*_2_ has significance differences with the other three distances. However, the median of the other three measurements has nonsignificance changes, and it can be assumed that the three distances has the same distribution. These conclusions are the same for the Experiment  2 distance measures.

Table 4 shows the average of the three distances (*d*_1_, *d*_3_, *d*_4_) as a global measure for both experiments. It represents the required correction when an augmented reality system is used and when it is not used.

## 4. Conclusions

This paper shows the protocol followed for the validation of an augmented reality system to help surgeons in the placement of trocars on patients in a real environment. First of all, the documentation that is normally required for patient involvement was presented to the hospital. The difference with other experiments, where new drugs or therapies are applicable, lies in the nonimpact that this process has on the patient or on the clinical staff because it does not introduce any additional risk to the surgery and no protocols are changed.

The hypothesis of this paper is that an augmented reality system can improve the placement of trocars in laparoscopic surgery. The results confirm this hypothesis since the average accuracy improved and its variability decreased when the AR system was used.

The Experiment  2 of our work was validated with a 3D model extracted from MR images. The reason for this modeling is that MR images are acquired in the normal protocol of the hospital where we performed the experiments for the laparoscopic cholecystectomies. However, the 3D model can be segmented from other types of images such as CT images. We used the navel as the anatomical structure of reference in the registration procedure of our AR system, but other external reference may be used for other surgeries if it is visible in the CT/MR images.

When Tables 1 and 2 are analyzed, the first trocar that is introduced in the patient (*d*_2_) shows a null improvement. This result is consistent since the surgeon has no additional information (i.e., laparoscopic camera view) to change the position since the first mark is drawn with the pen until the first incision is made as explained in the introduction; it is usually located one centimeter above the navel, so a minimal correction is made with or without the system.

As shown in Table 3, the system improved the trocar placement accuracy by 33%, while variability was reduced by 65%. The use of an augmented reality system can be helpful in complex situations by providing additional information, where even the use of an internal camera view is not enough for the required accuracy (and more incisions than necessary may be made as mentioned in Section 1). Another advantage of using the augmented reality system is its low cost and its applicability. When the surgeon has the internal information provided by the laparoscopic camera which has already been introduced into the patient, they have a finite and limited time to make the rest of the incisions. The longer the decision time, the higher the costs and the greater the risks for the patient. The augmented reality system is useful even in the hours before the operation (when the patient is awake and out of risk) making it possible to plan and reduce the time spent in the placement of the trocar in the operating theater. The augmented reality system also has direct application for automating and optimizing the trocar placement for guided surgery.

When (2) and (3) are analyzed, errors introduced by the augmented reality system (*d*_ra_, *d*_segment_, and *d*_other_) are much lower than the correction offered by the system (*d*_i_). The correction (in absolute value) achieved by the system in this work was on the order of *d* = 25 mm, while our AR algorithms introduced an error *d*_ra_ = 2.91 mm. If the correction or distance “*d*_*i*_” is the consequence of displacement produced by the technique of pneumoperitoneum and/or the subjectivity of the surgeon and/or the patient anatomical particularities, the system helps to correct the subjectivity and the particularities. The deformity and displacement that the pneumoperitoneum technique (*d*_neum_) produces could be solved if the 3D model was deformed the same way as the real deformations using a biomechanical or predictive model [36]. Since the augmented reality system offers an internal view of the patient's organs, it is hoped that the system can help to accurately determinate the displacement (*d*_patient_) that is currently corrected with the laparoscopic camera by introducing references that are not visible when the initial marks are made.

It is difficult to make a direct comparison with the literature results because different scenario particularities, the methods, the surgeries, and the type of “patient” involved in each validation (phantom, animals, or humans) as it was introduced in Section 1. Most of the authors validate their systems using numerical methods and/or using phantoms but few of them are evaluated in clinical settings with real patients, showing that the integration of augmented reality technology into the clinical environment and workflow is not common [9–15, 19–26]. It is often not feasible to evaluate a system based on surgical outcome or the impact of the system on the patient, but it is possible to evaluate these systems indirectly in phantoms and/or controlled environments [7]. The contribution of our work is that the system is validated and evaluated in a real environment with patients, and the benefits of using an AR system are demonstrated in a more realistic manner.

## Figures and Tables

**Table 1:** Advantages and disadvantages of different visualization devices.

Device	Advantages	Disadvantages
Projector	Direct vision of 3D model onto the patient, free movement of the surgeon in the scene, and easy hand-eye training.	Low resolution, ambient light in the operating theater that reduces projection sharpness, heat and air ventilation that are dangerous in sterile environments, shadow effect, and setup just above the scene that entails an associated risk.

AR glasses	High grade of immersion and good mobility of the surgeon in the scene but more reduced in comparison to the projector.	Possibility of dizziness, decrease of reality perception and loss of depth, additional material in the surgeon's field of view, and feeling of stress until surgeon gets used to it.

Monitor	Harmless for patient and surgeon, free movement in the scene (the screen can be placed farther from the doctor), easy to sterilize, and high resolution.	Hand-eye training since the surgeon must look up to see the monitor.

**Table 2:** Results of Experiment 1 (without the system).

	*d*_1_	*d*_2_	*d*_3_	*d*_4_
Average (cm)	1.13	0.13	1.88	1.00
Stan. deviat. (cm)	1.13	0.35	1.96	1.07

**Table 3:** Results of Experiment 2 (with the system).

	*d*_1_	*d*_2_	*d*_3_	*d*_4_
Average (cm)	**0.87**	**0.12**	**1.00**	**0.98**
Stan. deviat. (cm)	**0.53**	**0.35**	**0.53**	**0.35**

**Table 4:** Global result of the experiments.

	Without system	With system
Average (cm)	1.33	**0.87**
Stan. deviat. (cm)	1.43	**0.53**
